# BMI is associated with FEV_1_ decline in chronic obstructive pulmonary disease: a meta-analysis of clinical trials

**DOI:** 10.1186/s12931-019-1209-5

**Published:** 2019-10-29

**Authors:** Yilan Sun, Stephen Milne, Jen Erh Jaw, Chen Xi Yang, Feng Xu, Xuan Li, Ma’en Obeidat, Don D. Sin

**Affiliations:** 1 The Respiratory Department of the First Affiliated Hospital, College of Medicine, Zhejiang University, Hangzhou, Zhejiang Province China; 2 Centre for Heart Lung Innovation, St. Paul’s Hospital & Division of Respiratory Medicine, University of British Columbia, Rm 166-1081 Burrard Street, Vancouver, BC V6Z 1Y6 Canada; 3 Centre for Heart Lung Innovation, St. Paul’s Hospital & Department of Pathology and Laboratory Medicine, University of British Columbia, Vancouver, BC Canada

## Abstract

**Background:**

There is considerable heterogeneity in the rate of lung function decline in chronic obstructive pulmonary disease (COPD), the determinants of which are largely unknown. Observational studies in COPD indicate that low body mass index (BMI) is associated with worse outcomes, and overweight/obesity has a protective effect – the so-called “obesity paradox”. We aimed to determine the relationship between BMI and the rate of FEV_1_ decline in data from published clinical trials in COPD.

**Methods:**

We performed a systematic review of the literature, and identified 5 randomized controlled trials reporting the association between BMI and FEV_1_ decline. Four of these were included in the meta-analyses. We analyzed BMI in 4 categories: BMI-I (< 18.5 or <  20 kg/m^2^), BMI-II (18.5 or 20 to < 25 kg/m^2^), BMI-III (25 to < 29 or < 30 kg/m^2^) and BMI-IV (≥29 or ≥ 30 kg/m^2^). We then performed a meta-regression of all the estimates against the BMI category.

**Results:**

The estimated rate of FEV_1_ decline decreased with increasing BMI. Meta-regression of the estimates showed that BMI was significantly associated with the rate of FEV_1_ decline (linear trend *p* = 1.21 × 10^− 5^).

**Conclusions:**

These novel findings support the obesity paradox in COPD: compared to normal BMI, low BMI is a risk factor for accelerated lung function decline, whilst high BMI has a protective effect. The relationship may be due to common but as-of-yet unknown causative factors; further investigation into which may reveal novel endotypes or targets for therapeutic intervention.

## Background

Chronic obstructive pulmonary disease (COPD) is characterized by persistent and progressive airflow limitation, with significant variation in the rate of lung function decline between individuals [1]. Rapid lung function decline is associated with a number of factors including continued smoking, emphysema severity, and the frequency of acute exacerbations; identification and modification of such risk factors is a goal of COPD management [2]. However, the precise determinants of the rate of lung function decline are largely unknown.

Nutritional and metabolic abnormalities are core features of COPD as a systemic disease [3, 4]. Numerous observational studies have demonstrated that low BMI is an independent predictor of mortality in COPD [5–8]. Overweight and obesity, on the other hand, appear to have a protective effect on mortality, with the greatest effect in those with severe COPD [5, 6]. This is in contrast to the general population, where obesity is associated with decreased life expectancy [9]. Such observations form part of the so-called “obesity paradox” [10], but how this applies to other COPD outcomes is unclear. To determine the relationship between BMI and the rate of FEV_1_ decline in people with COPD, we performed a systematic review and a meta-regression of data from published randomized controlled trials. Combining data from over 27,000 clinical trial participants allowed us to explore the BMI-FEV_1_ decline relationship over a wide range of BMI and COPD severity.

## Methods

### Search strategy

This systematic review with meta-analysis is registered in the PROSPERO database [11] (registration number CRD42019118881) and performed according to the PRISMA guidelines [12]. We conducted a series of systematic searches in the PubMed, Embase and Cochrane Library databases (as of January 2019) using the search algorithm: (“pulmonary disease, chronic obstructive” [MeSH Terms] OR “COPD”) AND (FEV_1_ decline).

### Study selection

Two reviewers independently assessed the search results (including appendices, online supplements, and bibliographies) for eligibility. Studies were included if they met all of the following criteria: 1) conducted in participants with COPD, diagnosed by prespecified spirometric criteria 2) randomized controlled trial (RCT) design; 3) association between BMI and rate of decline in FEV_1_ reported; and 4) peer-reviewed publication. Studies were excluded if they met any of the following criteria: 1) participants with respiratory disorders other than COPD; 2) language other than English; or 3) non-original research publication (reviews, editorials, comments). The reviewers removed duplicates by identifying publications from the same research population, and retaining the publication with the highest quality score (see below).

### Data extraction and quality appraisal

Two reviewers independently appraised each study, with any discrepancies resolved by consensus, and extracted the relevant participant, protocol, and outcome data. The reviewers assessed the quality of included studies according to 7 assessment criteria in the Cochrane Collaboration Risk of Bias tool [13].

### Data analysis

For each of the included studies that specified subgroups based on a BMI interval, we extracted the mean rate of FEV_1_ decline and associated standard error for each BMI category. We then performed separate meta-analyses of FEV_1_ decline for each BMI category using a random-effects model, designating statistical significance as *p* < 0.05 (two-tailed). For each meta-analysis, we assessed for publication bias using funnel plots and an Egger’s test [14], and for heterogeneity using a Cochran’s Q test and the I^2^ statistic.

We then performed a meta-regression of the estimates using orthogonal polynomial regression (up to the third order, i.e. number of categories minus one), with BMI category as an ordinal variable, and the rate of FEV_1_ decline as a continuous variable. We assigned signficance to the model at *p* < 0.05.

## Results

### Search outcomes and study inclusion

**Fig. 1:**
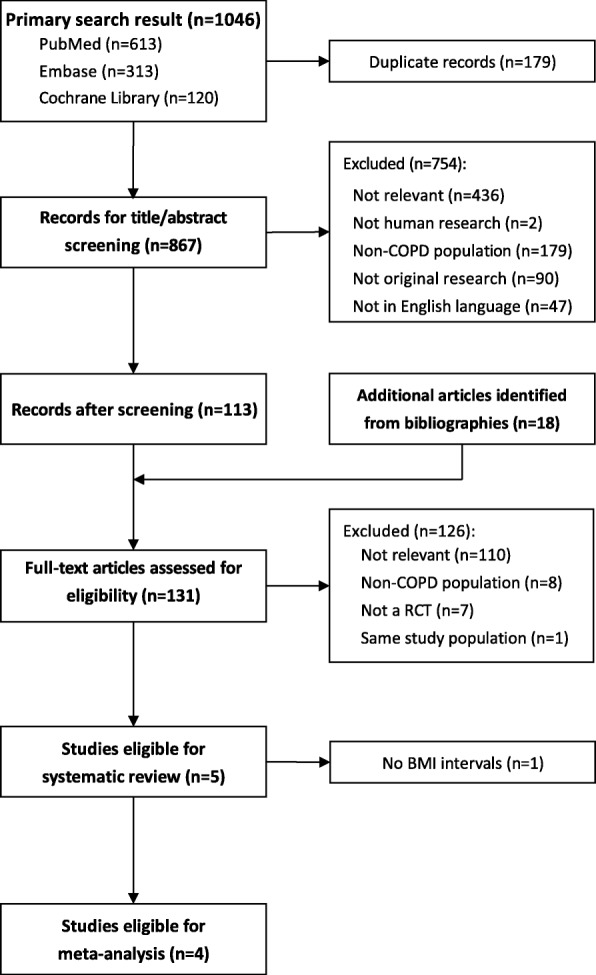
Workflow for systematic review. COPD, chronic obstructive pulmonary disease; RCT, randomized controlled trial; BMI, body mass index

A total of 5 studies satisfied the inclusion/exclusion criteria (Fig. 1). There was a broad range of sample sizes, study durations, and COPD severities. Detailed review of the individual study inclusion and exclusion criteria confirmed that there were no limits imposed on BMI for entry into the studies. The studies generally employed random or mixed modeling, but they differed by the coefficients included in the models. The study objectives varied considerably between the included studies. However, the BMI intervals employed for subgroup analysis were very similar (Table 1).

**Table 1:** Studies included in systematic review

Author (year of publication)	RCT source	Subjects (n)	Gender (M/F)	Age range (years)	COPD severity	%pred FEV_1_^a^	BMI (kg/m^2^)^b^	Duration (years)	Relevant study objectives
Celli (2008) [16]	TORCH	5343	4080/1263	40–80	Moderate-very severe (FEV_1_ ≤ 60%pred)	44.7 (13.1)	25.4 (5.2)	3	Effect of inhaled FP, and FP plus salmeterol on annualized rate of decline in post-bronchodilator FEV_1_, compared to placebo; prespecified subanalysis by BMI category
Calverley (2018) [17]	SUMMIT	15,457	11,559/3898	40–80	Moderate (FEV_1_ 50–70%pred)	59.7 (6.1)^c^	28.0 (6.0)	≥3	Effect of FF, VI, and FF plus VI on annualized rate of decline in post-bronchodilator FEV_1_, compared to placebo; prespecified subanalysis by BMI category
Anzueto (2015) [20]	TIOSPIR	1370	849/521	≥40	Moderate-very severe (FEV_1_ ≤ 70%pred)	47.5 (12.7)^d^	25.9 (5.1)^d^	2.3	Effect of TIO Respimat versus TIO HandiHaler on safety outcomes, with prespecified non-inferiority spirometry substudy examining annualized rate of decline in trough FEV_1_; prespecified subanalysis by BMI category
Tashkin (2008) [19]	UPLIFT	4964	3757/1207	≥40	Moderate-very severe (FEV_1_ ≤ 70%pred)	48.6 (13.4)	NS	4	Effect of TIO HandiHaler on annualized rate of decline in pre- and post-study drug FEV_1_, compared to placebo; prespecified subanalysis by BMI category within each treatment group
Tkacova (2016) [15]	LHS	5887	3702/2185	35–60	Mild-moderate (FEV_1_ 55–90%pred)	80^e^	25.2^e^	5	Effect of AHR on annualized rate of decline in FEV_1_ (unspecified bronchodilator), examining baseline BMI as a covariate

^a^pooled mean (pooled SD) of baseline post-bronchodilator FEV_1_%pred, unless otherwise stated. ^b^pooled mean (pooled SD) of baseline BMI unless otherwise stated ^c^from whole group data in original trial publication [18]. ^d^from whole group data (not specified for subgroup analysis). ^e^average of medians. %pred, percent predicted; *RCT* randomized controlled trial, *COPD* chronic obstructive puplmonary disease, *BMI* body mass index, *FEV*_*1*_ forced expiratory volume in 1 s, *FP* fluticasone propionate, *FF* fluticasone fuorate, *VI* vilanterol, *TIO* tiotropium, *AHR* airway hyperresponsiveness (measured by methacholine challenge test), *NS* not specified

### Quality appraisal

In general, the included studies were of good quality according to the Cochrane Collaboration tool (Additional file 1: Table S1). We designated 4 out of the 5 studies as low risk of bias based on all 7 criteria, and one study (Tkacova *et al* [15]) as high risk of bias based on a single criterion (“blinding of participants and personnel”).

### Outcomes of included studies

The extracted participant and study protocol data are shown in Table 1, and study outcomes are summarized in Table 2. Four of the 5 studies classified BMI at baseline into 4 intervals. The statistical methods of calculating FEV_1_ decline (e.g. model type and covariates) varied between the studies.

**Table 2:** Association of BMI with FEV_1_ decline in the systematically reviewed studies

Author (year of publication)	RCT source	Statistical method	BMI association with FEV_1_ decline^a^		Significance	Nature of relationship
Celli (2008) [16]	TORCH	Random coefficients model. Covariates: baseline FEV_1_, age, sex, smoking status, treatment, time on treatment, treatment*time, region	BMI interval, kg/m^2^	FEV_1_ decline (SE), mL/yr	Effect of BMI on FEV_1_ decline, *p* < 0.001	FEV_1_ decline decreases with increasing BMI
			< 20 (*n* = 719)	−51.1 (4.4)		
			20 to < 25 (*n* = 2003)	−50.5 (2.5)		
			25 to < 29 (*n* = 1424)	−42.1 (2.9)		
			≥29(*n* = 1197)	−35.1 (3.2)		
Calverley (2018) [17]	SUMMIT	Random coefficients model. Covariates: baseline FEV_1_, age, sex, smoking status, treatment, time, treatment*time	BMI interval, kg/m^2^	FEV_1_ decline (SE), mL/yr	Effect of BMI on FEV_1_ decline, *p* < 0.001	FEV_1_ decline decreases with increasing BMI
			< 18.5 (*n* = 494)	−52 (7.2)		
			18.5 to < 25 (*n* = 4562)	−50 (2.3)		
			25 to < 30 (*n* = 5362)	−40 (2)		
			≥30 (*n* = 5039)	−37 (2.1)		
Tashkin (2008) [19]	UPLIFT	Random coefficients model. Covariates: treatment, subgroup, subgroup*BMI	BMI interval, kg/m^2^	FEV_1_ decline (SE),mL/yr	ND	FEV_1_ decline decreases with increasing BMI in both treatment groups
			Tiotropium			
			< 20 (*n* = 242)	−53 (4)		
			20 to < 25 (*n* = 915)	−44 (2)		
			25 to < 30 (*n* = 903)	−36 (2)		
			≥30(n = 494)	−34 (3)		
			Placebo			
			< 20 (*n* = 280)	−55 (4)		
			20 to < 25 (*n* = 782)	−49 (2)		
			25 to < 30 (*n* = 853)	−37 (2)		
			≥30 (*n* = 495)	−34 (3)		
Anzueto (2015) [20]	TIOSPIR	Mixed repeated measures model. Covariates: treatment, visit, treatment*visit as fixed effects, with random intercept and slope	BMI interval, kg/m^2^	FEV_1_ decline (SE), mL/yr^b^	ND	Inverse J-shape – lowest FEV_1_ decline in overweight (BMI 25 to < 30 kg/m^2^)
			< 18.5 (*n* = 51)	−45.2 (19.9)		
			18.5 to < 25 (*n* = 436)	−32.1 (6.6)		
			25 to < 30 (*n* = 492)	−31.8 (6.1)		
			≥30 (*n* = 391)	−35.5 (6.8)		
Tkacova (2016) [15]	LHS	Multivariate linear model. Covariates: baseline FEV_1_, age, sex, BMI, smoking status	Coefficient of BMI not significant, *p* = 0.489			No significant association

^a^Change in post-bronchodilator or post-study drug measurements unless otherwise stated. ^b^change in trough FEV_1_. RCT, randomized controlled trial; *BMI* body mass index, *FEV*_*1*_ forced expiratory volume in 1 s, *SE* standard error, *ND*, not determined

The study by Celli *et al* [16] was a post hoc analysis of the Toward a Revolution in COPD Health (TORCH) study, which was a multi-center, randomized, double-blind and placebo-controlled trial of fluticasone propionate with or without salmeterol treatment in participants with moderate to severe COPD. The pooled mean (pooled SD) post-bronchodilator FEV_1_ (30 min following 400 μg albuterol) was 44.7 (13.1) percent predicted (%pred), and the pooled mean BMI was 25.4 (5.2) kg/m^2^. The rate of post-bronchodilator FEV_1_ decline decreased as BMI interval increased.

Calverley *et al* [17] explored the effect of baseline BMI on lung function decline as secondary outcome analysis of the Study to Understand Mortality and Morbidity (SUMMIT) trial of fluticasone fuorate and vilanterol in patients with moderate COPD and a history, or increased risk of, cardiovascular disease. Data on the mean FEV_1_%pred were not available for this analysis. In the original trial publication [18], the pooled mean post-bronchodilator FEV_1_ (30 min following 400 μg albuterol) was 59.7 (6.1) %pred. In the secondary outcome analysis, the pooled mean BMI was 25 (6) kg/m^2^.The rate of post-bronchodilator FEV_1_ decline decreased with increasing BMI interval.

The study reported by Tashkin *et al* [19] was a subanalysis of the Understanding Potential Long-term Impacts of Function with Tiotropium (UPLIFT) trial, which was a randomized, double-blind and placebo-controlled trial of tiotropium in participants with moderate to severe COPD. The mean %pred FEV_1_ and mean BMI for subjects included in the subanalysis were not specified; however, for the whole group, the pooled mean post-bronchodilator (90 min following 80 μg ipratropium and 30 min following 400 μg albuterol) FEV_1_ was 47.5 (12.7) %pred and pooled mean BMI was 25.9 (5.1) kg/m^2^. The authors reported the rate of FEV_1_ decline for each BMI category separately for the tiotropium and placebo groups; in both groups, the annualized rate of post-bronchodilator (study drug, ipratropium and albuterol) FEV_1_ decline tended to decrease with increasing BMI category.

Anzueto *et al* [20] reported on a prespecified spirometry sub-study of the Tiotropium Safety and Performance in Respimat (TIOSPIR) trial, which was designed to assess the safety and efficacy of two different formulations of tiotropium in participants with moderate to very severe COPD. The pooled mean post-bronchodilator (drug and timing not specified) FEV_1_ was 48.6 (13.4) %pred. The mean BMI of subjects in this spirometry substudy was not specified, and since the subjects represented a small fraction of the entire study subjects, the whole group BMI could not be generalized. For the relationship between BMI interval and FEV_1_ decline, the publication provided only a Forest plot. We therefore contacted the corresponding author, who kindly provided the numerical data. The mean annualized rate of trough (pre-medication) FEV_1_ decline was greatest in the lowest BMI category, with negligible differences between the two middle categories. However, unlike the previous studies, the rate of trough FEV_1_ decline was slightly greater in the highest BMI category compared to the middle categories.

Finally, Tkacova *et al* [15] reported on a post hoc analysis of data from the Lung Health Study (LHS), which was a 5 year, randomized, placebo-controlled trial in smokers with mild-moderate COPD. Participants were allocated to standard care or a smoking cessation intervention, with or without ipratropium bromide. For this study, the primary analysis was the relationship between airway hyperresponsiveness (AHR) and FEV_1_ decline. Baseline FEV_1_ and BMI were reported as median (interquartile range) for subjects grouped according to AHR status; the average of medians for post-bronchodilator (“two puffs” of isoproterenol) FEV_1_ was 80%pred, and the average of medians for BMI was 25.2 kg/m^2^. The authors did not categorize participants into BMI intervals. However, in a multivariable linear model, the effect of BMI as a continuous variable on annualized post-bronchodilator FEV_1_ decline was not significant.

### Meta-analysis

We excluded the study by Tkacova et al (LHS) [15] from the meta-analysis due to the absence of a BMI interval subgroup analysis. In the remaining studies, the specified BMI intervals were quite similar (Table 2). Therefore, for the purposes of the meta-analyses, we treated the corresponding BMI categories as equivalent, relabeling them (from lowest to highest) as BMI- I, BMI-II, BMI-III and BMI-IV. Since the estimates in the study by Tashkin et al (UPLIFT) [19] were reported separately for placebo and treatment groups, we first combined these estimates by meta-analysis to calculate whole-group estimates for each BMI category. The proportions of subjects in each category were: BMI-I, 7%; BMI-II, 32%; BMI-III, 33%; BMI-IV, 28%.

**Fig. 2:**
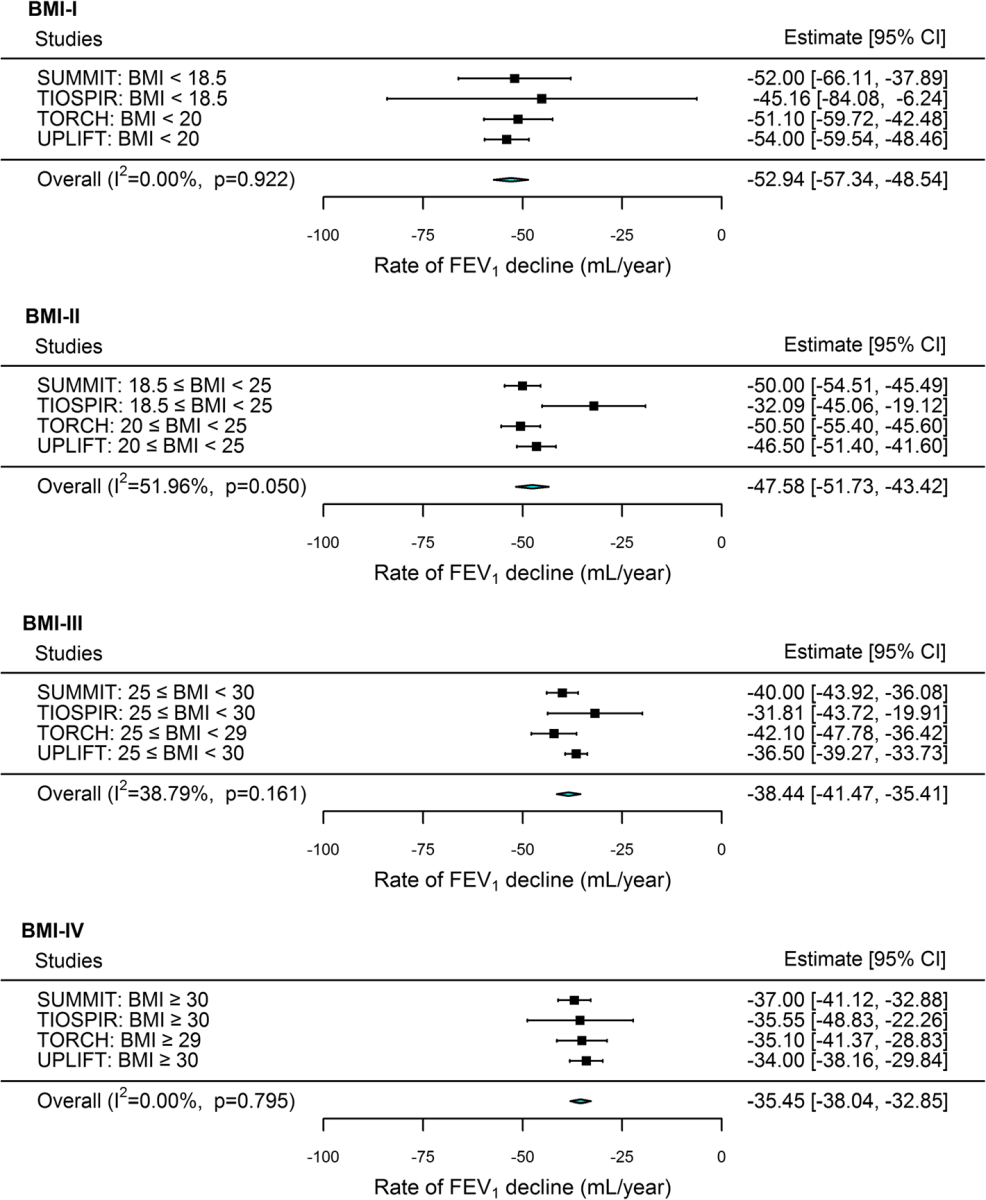
Meta-analyses of annualized rate of FEV_1_ decline by body mass index (BMI) category. Individual meta-analyses presented for each BMI category from lowest (BMI-I) to highest (BMI-IV). Data from randomized controlled trials: SUMMIT, Calverley et al [17]; TIOSPIR, Anzueto et al [20]; TORCH, Celli et al [16]; UPLIFT, Tashkin et al. [19] FEV_1_, forced expiratory volume in 1 s; CI, confidence interval; I^2^, heterogeneity statistic; p, significance from Cochran’s Q test of heterogeneity

The estimated annual rate of FEV_1_ decline decreased with increasing BMI category (Fig. 2): annual rate (95% confidence interval) for BMI-I, − 52.9 (− 57.3 to − 48.5) mL/yr; BMI-II, − 47.58 (− 51.7 to − 43.4) mL/yr; BMI-III, − 38.4 (− 41.5 to − 35.4) mL/yr; BMI-IV, − 35.5 (− 38 to − 32.9) mL/yr. Cochran’s Q tests and I^2^ values < 50% suggested low heterogeneity for all but the BMI-II meta-analysis. Funnel plots (Additional file 1: Figure S1) demonstrated significant asymmetry in the BMI-II category (Egger’s test *p* = 0.01), suggesting publication bias [14] driven by the study by Anzueto et al (TIOSPIR) [20]. When we excluded this study in a sensitivity analysis, the overall results for each meta-analysis were unchanged (Additional file 1: Figures S2 and S3).

**Fig. 3:**
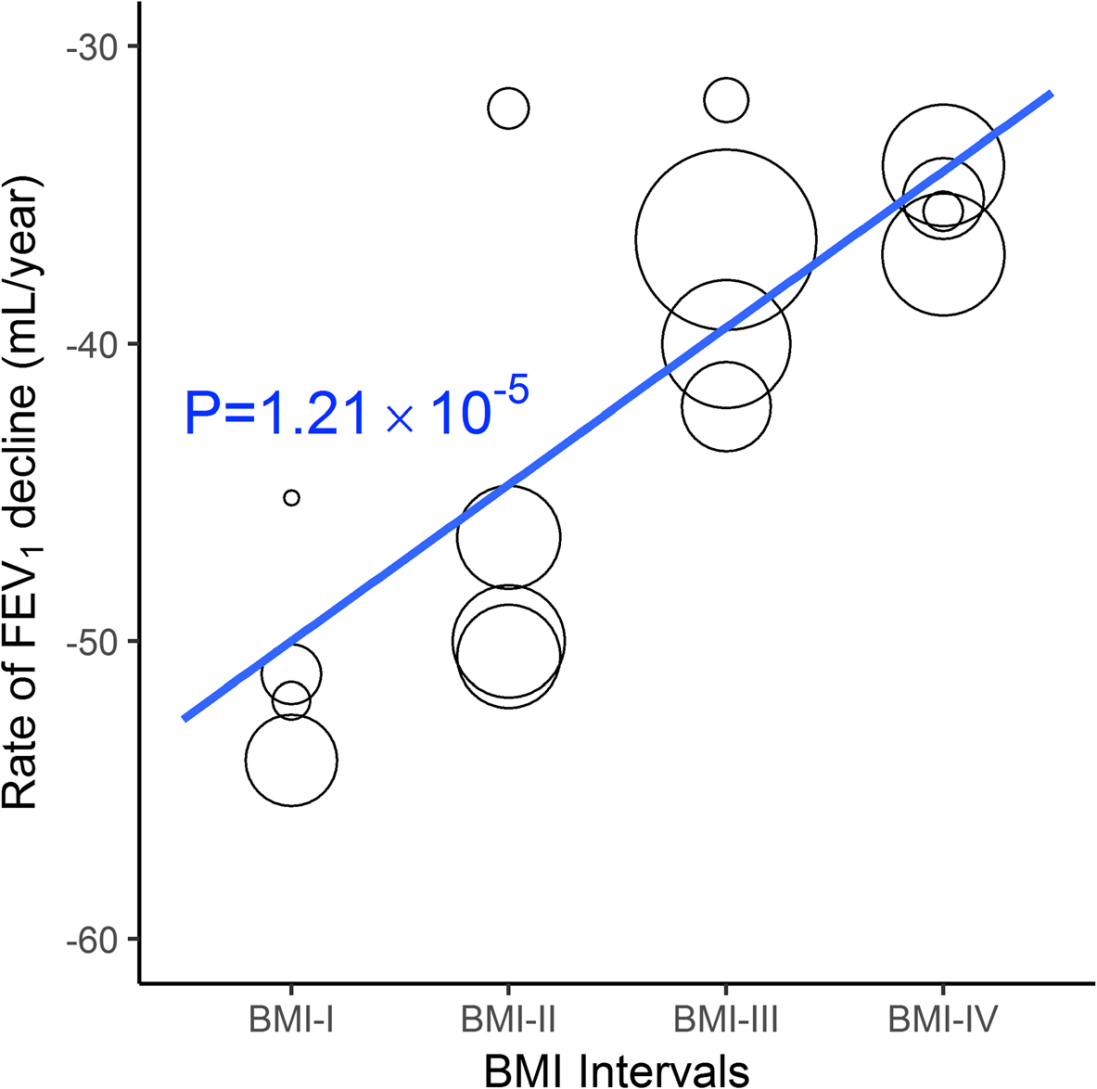
Meta-regression of annualized rate of FEV_1_ decline by body mass index (BMI). Inverse variance weighted (IVW) model, with BMI as an ordinal variable from BMI-I (lowest) to BMI-IV (highest). Size of circles represents relative weighting of estimates. See main text from description of BMI categories. FEV_1_, forced expiratory volume in 1 s; p, significance of linear trend

Meta-regression of the estimates showed a significant linear trend between BMI category and the annual rate of FEV_1_ decline (adjusted R^2^ = 0.81, *p* = 1.2 × 10^− 5^) (Fig. 3). Second- and third-order regression estimates were not significant. A significant linear trend remained following sensitivity analysis (exclusion of the study by Anzueto et al (TIOSPIR) [20]) (adjusted R^2^ = 0.9, *p* = 3 × 10^− 5^); for this model, the cubic trend was also significant (*p* = 0.03) (Additional file 1: Figure S4).

## Discussion

Using a systematic review and meta-regression of published RCT results, we have shown that BMI is significantly associated with the rate of lung function decline in COPD. Compared to normal BMI, lower BMI was associated with a faster FEV_1_ decline. This phenomenon is common to other chronic respiratory conditions such as cystic fibrosis [21], as well as the general population [22]. Additionally, higher BMI was associated with slower FEV_1_ decline. In fact, the mean rate of FEV_1_ decline in the obese category (BMI-IV, − 35 mL/yr) was not too dissimilar from some estimates in the non-smoking, non-COPD population [23]. This finding adds to the existing body of literature suggesting that low BMI is a risk factor for poor outcomes in COPD, and that increased BMI has a protective effect.

Our systematic search strategy found 5 good-quality studies meeting the prespecified inclusion criteria, 4 of which reported subgroup analysis by BMI intervals that were largely similar between the studies. This allowed us to combine the results within each BMI category for meta-analysis and subsequent meta-regression of the estimates. Only one category (BMI-II) showed evidence of a publication bias, and sensitivity analysis suggests that this had neglibigle impact on the overall result. There was heterogeneity amongst the included studies in terms of the covariates used in the statistical models – most notably, whether the models accounted for baseline FEV_1_ and smoking status. Both of these factors are known to influence the rate of decline in FEV_1_, although the extent to which this heterogeneity influences the overall meta-regression cannot be determined from the summary data available to us. The proportion of subjects in the obese (BMI-IV) category was 28%, which is lower than the estimates of obesity prevalence in both the general population (36% in the USA) [24] and in COPD cohort studies (35% in the COPDGene cohort) [25]. The reason for, and impact of, this discrepancy is unclear.

BMI encompasses both fat mass and muscle mass, but the relative contributions of these body compartments to the BMI-FEV_1_ decline relationship cannot be determined from our data. The distinction is important, since the fat-free mass index (FFMI) – which reflects nutritional state – is also independently and inversely-correlated with mortality in both general [26, 27] and COPD [28] populations. In COPD, low FFMI is associated with more severe lung function impairment and reduced exercise capacity [29–31], however its impact on lung function decline over time is not known.

A possible explanation for the inverse correlation between BMI and lung function decline is “reverse-causation”, where increased COPD disease activity or severity leads to weight loss and cachexia. Postulated mechanisms include increased resting energy consumption [32], non-respiratory skeletal muscle atrophy due to decreased peripheral oxygen availability and disuse [33, 34], and systemic inflammation [35]. In this scenario, the BMI-FEV_1_ decline relationship should plateau in the normal BMI range, or even form a U-shaped curve (extrapolating findings from general population studies) [36, 37]. Instead, we found a clear linear trend such that overweight/obese subjects had the slowest rate of FEV_1_ decline – a finding consistent with the “obesity paradox”. Two of the 4 studies included in the meta-analysis accounted for disease severity by adjusting lung function decline for baseline FEV_1_, although this may not adequately account for any non-linearity of FEV_1_ decline as a function of disease severity. Other important aspects of disease activity, such as emphysema burden or exacerbation frequency, were not accounted for by any of the studies. Reverse-causality could be further explored by stratifying the analysis by GOLD stages, or other surrogates measures of disease severity. It would be particularly pertinent to examine the effect of cachexia, which can be present despite a high BMI, since this may also be a sign of increased disease activity. This would require other measures of nutritional status (such as FFMI) and spontaneous weight loss history. However, with only summary data available to us, these more detailed analyses were not possible, and reverse causation due to high disease activity remains a possible explanation for our findings.

An alternative explanation is that BMI is causally related to lung function decline. There is some evidence that extreme weight loss can induce lung damage, with anorexia nervosa patients [38] and animal models of starvation [39, 40] showing signs of early emphysema. Once again, these observations may explain the low BMI association, but do not adequately explain the apparent protective effect of obesity over normal BMI demonstrated by our results.

Another explanation is that common factors modulate both BMI and lung function decline in COPD. The phenotype may even be under genetic control. In combined COPD cohorts, Wan *et al* [41] found a significant association between BMI and a variant in the fat mass and obesity-associated (FTO) gene (which has strong and reproducible associations with BMI in almost all general population studies). The rs8050136 minor allele was associated with higher BMI, better lung function, and a lower emphysema score. Using the known genetic architecture of BMI in adequately-powered studies in COPD subjects may help to explore this observation. Physical activity is one factor associated with both BMI and lung function decline [42], although there is limited evidence that exercise interventions such as pulmonary rehabilitation have any measurable impact on change in FEV_1_ over time [43]. Nevertheless, the potential long-term effects of physical activity on FEV_1_ decline for a given BMI should be taken into account in future studies.

The adipokines, tumor necrosis factor-alpha (TNF-α) and interleukin-6 (IL-6), have also been implicated in both body mass regulation and COPD pathogenesis. TNF-α and IL-6 have pleiotropic effects including regulation of lung inflammation, lipolysis, and skeletal muscle atrophy [44, 45], and IL-6 knockout mice develop obesity [46]. In the context of the obesity paradox, it is possible that differential expression and/or sensitivity to these pathways give rise to a spectrum of phenotypes in COPD, with low BMI/rapid lung function decline at one end, and high BMI/slow lung function decline at the other. The systemic effects of TNF-α and IL-6 may be modulated by circulating soluble receptors [47, 48], or other adipokines such as adiponectin. Summer *et al* [49] showed that adiponectin-deficient mice had higher lung TNF-α expression, and developed weight loss and an emphysema-like phenotype. This is in contrast to observations in humans, where high serum adiponectin levels were associated with increased respiratory mortality and accelerated FEV_1_ decline [50]. Nevertheless, a “favorable” adipokine profile may explain the apparent protective effect of high versus normal BMI on lung function decline, and vice versa.

Pneumoproteins such as surfactant protein-D (SP-D), which is secreted by Type II alveolar cells and is a part of the lung’s innate immune response [51], may also modulate both lung function and BMI. SP-D is decreased in the lungs of current and former smokers, and may have a causal role in COPD pathogenesis and progression [52]. SP-D deficiency is associated with obesity in human population studies [53], and SP-D-knockout mice develop obesity [54] as well as emphysema [55]. Conversely, circulating SP-D may induce lipolysis and weight loss via the IL-6 pathway [54]. The systemic effects (e.g. body weight regulation, or risk of comorbidities) of SP-D and other pneumoproteins warrant further investigation, since it is likely that a complex interaction of pathways is at play.

Our analysis has several limitations. Firstly, we chose to review only FEV_1_ decline as the outcome. Although FEV1 decline is an important prognostic marker (and an endpoint recognized by regulatory agencies such as the FDA), the effects of BMI on other longitudinal outcomes such as exacerbation rate, health care utilization, change in exercise capacity (e.g. 6-min walk test distance), and symptoms warrant similar investigation. Secondly, we included only published, English-language studies, which may be an important source of bias. Thirdly, we included only RCTs (and not observational studies) in order to have well-defined and relatively homogenous study populations, with strict standardizations of follow-up and quality control of spirometric measurements. This led to only a small number of studies being included. However, the fact all studies except TIOSPIR by Anzueto *et al* [20] showed a consistent direction of effect of BMI on FEV_1_ decline, and that they examined a combined population of over 27,000 participants, means there was sufficient statistical power for a highly-significant meta-regression estimate. Finally, we had access only to summary statistics from each trial, which restricted our meta-analyses to pre-defined BMI categories and led to the exclusion of one study [15]. Since the excluded study found no significant effect of BMI on FEV_1_ decline in a multivariable model, this may have biased our result in favor of an association. Access to individual-level data would allow us to model the BMI-FEV_1_ decline relationship under different assumptions such as nonlinearity, which may be relevant for the effect of baseline lung function, and also at the extremes of BMI where any “dose effect” of obesity could be determined. Analysis could also be stratified by disease severity, gender, smoking intensities, and the use of medications such as inhaled corticosteroids. Exacerbation history may also be examined as a potential modifying factor, since there are conflicting data on the effect of obesity on exacerbation risk [25, 56]. Individual-level data would also allow us to apply a consistent statistical model for determining FEV_1_ decline, thus addressing the heterogeneity of modeling amongst the included studies.

## Conclusions

Through systematic review and meta-regression of RCT data, we have demonstrated that BMI is associated with the rate of lung function decline in COPD. Compared to normal BMI, low BMI is associated with faster, and high BMI is associated with slower, FEV_1_ decline. This finding is consistent with the “obesity paradox”. The mechanisms are likely complex, and further investigation of potential causative factors may yield novel endotypes, or unique pathways for therapeutic intervention.

## Supplementary information

**Additional file 1: Table S1.** Quality appraisal of studies in systematic review. **Figure S1.** Funnel plots and Egger’s test for publication bias for meta-analyses of annualized rate of FEV_1_ decline by body mass index (BMI) category. **Figure S2.** Meta-analyses of annualized rate of FEV_1_ decline by body mass index (BMI) category, following sensitivity analysis. **Figure S3.** Funnel plots and Egger’s test for publication bias for meta-analyses of annualized rate of FEV_1_ decline by body mass index (BMI) category, following sensitivity analysis. **Figure S4.** Meta-regression of annualized rate of FEV_1_ decline by body mass index (BMI), following sensitivity analysis.

## Data Availability

Not applicable

## Abbreviations

BMI: Body mass index
COPD: Chronic obstructive pulmonary disease
FEV_1_: Forced expiratory volume in 1 s
FFMI: Fat-free mass index
IL-6: Interleukin-6
LHS: Lung Health Study
RCT: Randomized controlled trial
SP-D: Surfactant protein-D
TNF-α: Tumor necrosis factor-alpha
