# Supplementary material for: BMI is associated with FEV1 decline in chronic obstructive pulmonary disease: a meta-analysis of clinical trials
Source: Respir Res. 2019 Oct 29;20:236. doi: 10.1186/s12931-019-1209-5 (PMC6819522; doi:10.1186/s12931-019-1209-5)
Supplement: Supplementary file 1 — Additional file 1: Table S1. Quality appraisal of studies in systematic review. Figure S1. Funnel plots and Egger’s test for publication bias for meta-analyses of annualized rate of FEV1 decline by body mass index (BMI) category. Figure S2. Meta-analyses of annualized rate of FEV1 decline by body mass index (BMI) category, following sensitivity analysis. Figure S3. Funnel plots and Egger’s test for publication bias for meta-analyses of annualized rate of FEV1 decline by body mass index (BMI) category, following sensitivity analysis. Figure S4. Meta-regression of annualized rate of FEV1 decline by body mass index (BMI), following sensitivity analysis. [file 12931_2019_1209_MOESM1_ESM.docx]

ONLINE SUPPLEMENTARY MATERIAL

ACCOMPANYING MANUSCRIPT TITLE:

BMI is associated with FEV_1_ decline in chronic obstructive pulmonary disease: a meta-analysis of clinical trials

AUTHORS:

Yilan Sun, Stephen Milne, Jen Erh Jaw, Chen Xi Yang, Feng Xu, Xuan Li, Ma'en Obeidat, Don D. Sin

CORRESPONDING AUTHOR:

Don D. Sin, MD

Room 548, Burrard Building, St. Paul’s Hospital, Vancouver, BC, Canada, V6Z 1Y6

Email: [don.sin@hli.ubc.ca](mailto:don.sin@hli.ubc.ca)

Voice: 1-604-806-8395

FAX: 1-604-806-9274

**CONTENTS:**

| **Table S1** | Quality appraisal of studies in systematic review |
| --- | --- |
| **Figure S1** | Funnel plots and Egger’s test for publication bias for meta-analyses of annualized rate of FEV_1_ decline by body mass index (BMI) category |
| **Figure S2** | Meta-analyses of annualized rate of FEV_1_ decline by body mass index (BMI) category, following sensitivity analysis |
| **Figure S3** | Funnel plots and Egger’s test for publication bias for meta-analyses of annualized rate of FEV_1_ decline by body mass index (BMI) category, following sensitivity analysis |
| **Figure S4** | Meta-regression of annualized rate of FEV_1_ decline by body mass index (BMI), following sensitivity analysis |
| **References** |  |

| **Author (year of publication)** | **Random sequence generation** | **Allocation concealment** | **Blinding of participants and personnel** | **Blinding of outcome assessment** | **Incomplete outcome data** | **Selective reporting** | **Other bias** |
| --- | --- | --- | --- | --- | --- | --- | --- |
| Celli (2008)^1^ | Low risk | Low risk | Low risk | Low risk | Low risk | Low risk | Low risk |
| Calverley (2018)^2^ | Low risk | Low risk | Low risk | Low risk | Low risk | Low risk | Low risk |
| Tashkin (2008)^3^ | Low risk | Low risk | Low risk | Low risk | Low risk | Low risk | Low risk |
| Anzueto (2015)^4^ | Low risk | Low risk | Low risk | Low risk | Low risk | Low risk | Low risk |
| Tkacova (2016)^5^ | Low risk | Low risk | ***High* *risk*** | Low risk | Low risk | Low risk | Low risk |

**Table S1**

**Quality appraisal of studies in systematic review.** Risk of bias categorized using Cochrane Collaboration tool [12]

**Figure S1**


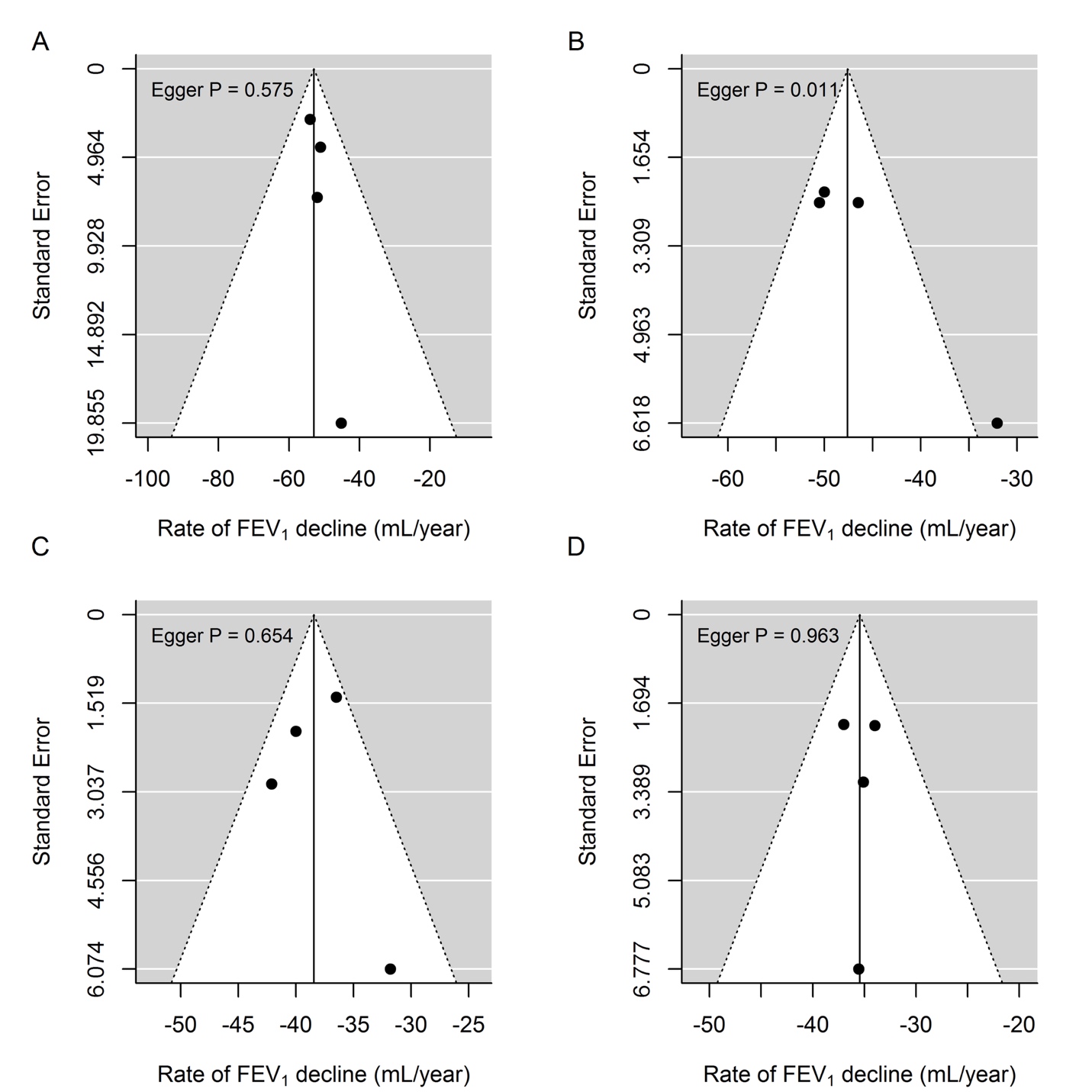


**Funnel plots and Egger’s test for publication bias for meta-analyses of annualized rate of FEV_1_ decline by body mass index (BMI) category.** (A-D), categories BMI-I to BMI-IV, respectively. See main text for description of BMI categories. FEV_1_, forced expiratory volume in 1 s.

**Figure S2**


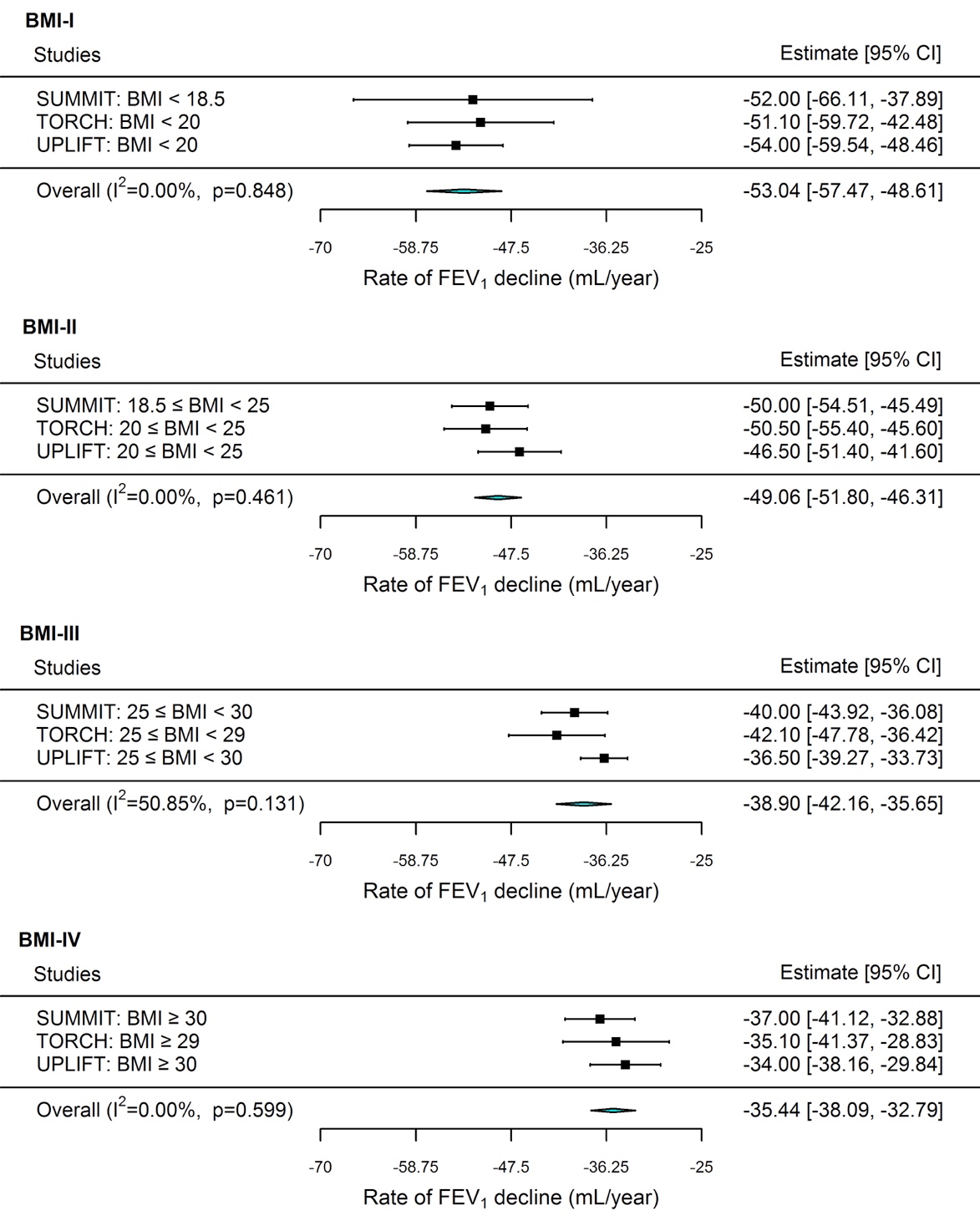


**Meta-analyses of annualized rate of FEV_1_ decline by body mass index (BMI) category, following sensitivity analysis.** For this sensitivity analysis, data from TIOSPIR (Anzueto *et al^4^*) was removed from the main meta-analysis due to evidence of publication bias. Individual meta-analyses presented for each BMI category from lowest (BMI-I) to highest (BMI-IV). Data from randomized controlled trials: SUMMIT, Calverley *et al*;^2^ TORCH, Celli *et al*;^1^ UPFLIFT; Tashkin *et al*.^3^ FEV_1_, forced expiratory volume in 1 s; CI, confidence interval; I^2^, heterogeneity statistic; p, significance from Cochran’s Q test of heterogeneity.

**Figure S3**

**
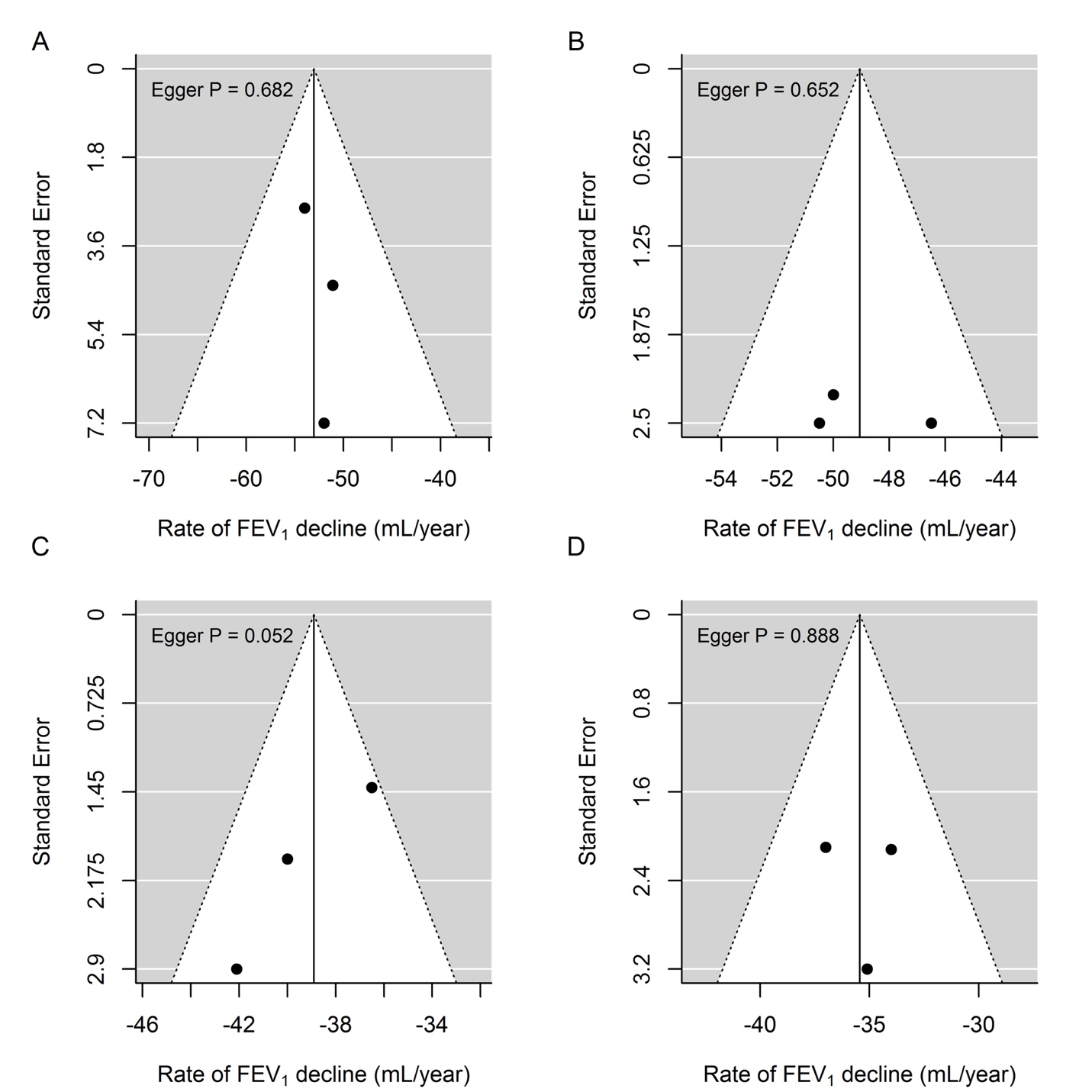
**

**Funnel plots and Egger’s test for publication bias for meta-analyses of annualized rate of FEV_1_ decline by body mass index (BMI) category, following sensitivity analysis.** For this sensitivity analysis, data from TIOSPIR (Anzueto *et al*^4^) was removed from the main meta-analysis due to evidence of publication bias. (A-D), categories BMI-I to BMI-IV, respectively. See main text for description of BMI categories. FEV_1_, forced expiratory volume in 1 s.

**Figure S4**


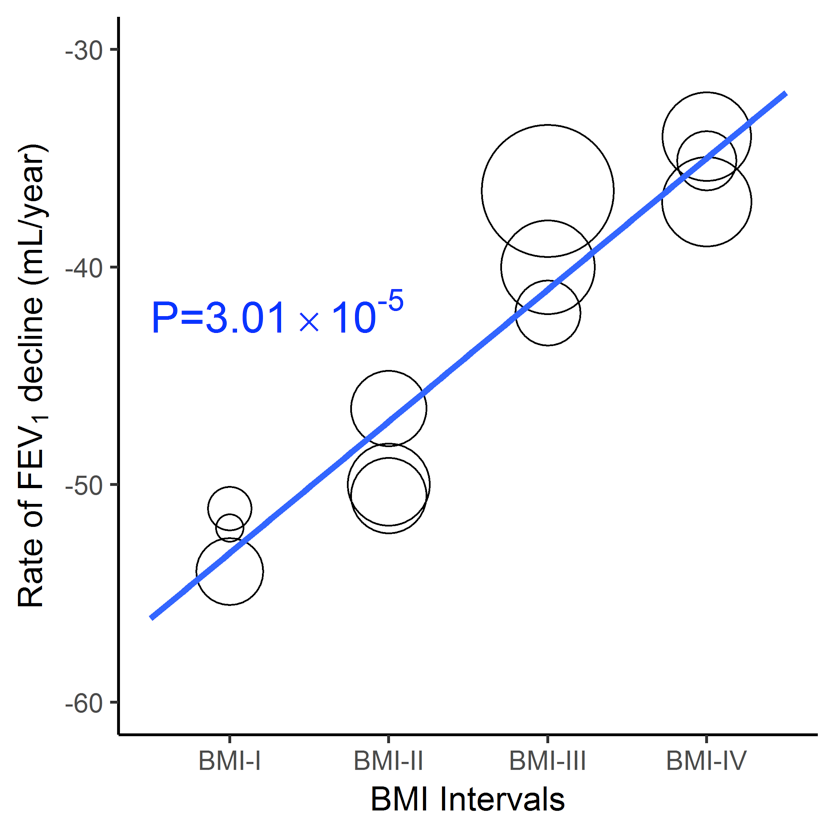


**Meta-regression of annualized rate of FEV_1_ decline by body mass index (BMI), following sensitivity analysis.** For this sensitivity analysis, data from TIOSPIR (Anzueto *et al*^4^) was removed from the main meta-analysis due to evidence of publication bias. Inverse variance weighting (IVW) model with BMI as a continuous variable from BMI-I (lowest) to BMI-IV (highest). See main text from description of BMI categories. Size of circles represents relative weighting of estimates. FEV_1_, forced expiratory volume in 1 s; p, significance of linear trend

**References**

1. Celli BR, Thomas NE, Anderson JA*, et al.* Effect of pharmacotherapy on rate of decline of lung function in chronic obstructive pulmonary disease: results from the TORCH study. *Am J Respir Crit Care Med.* 2008;178(4):332-338.

2. Calverley PMA, Anderson JA, Brook RD*, et al.* Fluticasone Furoate, Vilanterol, and Lung Function Decline in Patients with Moderate Chronic Obstructive Pulmonary Disease and Heightened Cardiovascular Risk. *Am J Respir Crit Care Med.* 2018;197(1):47-55.

3. Tashkin DP, Celli B, Senn S*, et al.* A 4-year trial of tiotropium in chronic obstructive pulmonary disease. *N Engl J Med.* 2008;359(15):1543-1554.

4. Anzueto A, Wise R, Calverley P*, et al.* The Tiotropium Safety and Performance in Respimat(R) (TIOSPIR(R)) Trial: Spirometry Outcomes. *Respir Res.* 2015;16:107.

5. Tkacova R, Dai DLY, Vonk JM*, et al.* Airway hyperresponsiveness in chronic obstructive pulmonary disease: A marker of asthma-chronic obstructive pulmonary disease overlap syndrome? *J Allergy Clin Immunol.* 2016;138(6):1571-1579 e1510.
